# Enhanced Osteogenic Differentiation of Human Fetal Cartilage Rudiment Cells on Graphene Oxide-PLGA Hybrid Microparticles

**DOI:** 10.3390/jfb10030033

**Published:** 2019-07-30

**Authors:** Stuart C. Thickett, Ella Hamilton, Gokulan Yogeswaran, Per B. Zetterlund, Brooke L. Farrugia, Megan S. Lord

**Affiliations:** 1 School of Natural Sciences (Chemistry), University of Tasmania, Hobart, TAS 7001, Australia; 2 Graduate School of Biomedical Engineering, UNSW Sydney, Sydney, NSW 2052, Australia; 3 Centre for Advanced Macromolecular Design, School of Chemical Engineering, UNSW Sydney, Sydney, NSW 2052, Australia

**Keywords:** PLGA, graphene oxide, osteoblast, stem cell, bone tissue engineering

## Abstract

Poly(d,l–lactide–co–glycolide) (PLGA) has been extensively explored for bone regeneration applications; however, its clinical use is limited by low osteointegration. Therefore, approaches that incorporate osteoconductive molecules are of great interest. Graphene oxide (GO) is gaining popularity for biomedical applications due to its ability to bind biological molecules and present them for enhanced bioactivity. This study reports the preparation of PLGA microparticles via Pickering emulsification using GO as the sole surfactant, which resulted in hybrid microparticles in the size range of 1.1 to 2.4 µm based on the ratio of GO to PLGA in the reaction. Furthermore, this study demonstrated that the hybrid GO-PLGA microparticles were not cytotoxic to either primary human fetal cartilage rudiment cells or the human osteoblast-like cell line, Saos-2. Additionally, the GO-PLGA microparticles promoted the osteogenic differentiation of the human fetal cartilage rudiment cells in the absence of exogenous growth factors to a greater extent than PLGA alone. These findings demonstrate that GO-PLGA microparticles are cytocompatible, osteoinductive and have potential as substrates for bone tissue engineering.

## 1. Introduction

The repair of critical size bone defects as a result of trauma, tumor resection or congenital abnormalities is an unmet clinical need for orthopedics and dentistry. While bone grafts are the clinical gold standard, their limitations include a restricted supply and require a secondary surgical site. Thus, there is interest in developing off-the-shelf biomaterial strategies for rapid bone repair. Biodegradable synthetic polymers such as poly(lactide–co–glycolide) (PLGA) have been widely explored for bone tissue engineering applications due to their tunable mechanical and degradation properties, unlimited availability and approved clinical use by the US Food and Drug Administration [1,2,3]. While PLGA has many attractive features for tissue engineering, it exhibits a poor osteoconductivity necessitating its complexation with osteoconductive molecules to achieve bone repair in clinically useful time frames [4].

Graphene oxide (GO) has gained attention for biomedical applications due to its cytocompatibility and capacity to bind various biological molecules via its surface carbon and oxygen functional groups, which also stabilize the GO sheets in aqueous media [5,6]. The range of functional groups in GO enables it to be readily incorporated into a polymer matrix and often results in improved mechanical and biological properties compared to the neat polymer [7]. Indeed GO-PLGA microparticles alone or incorporated into silk membranes have been reported to support osteoblast growth over 28 days with the addition of exogenous growth factors [8,9,10,11].

PLGA particles can be prepared by a range of methods including emulsion-evaporation, emulsion-diffusion, “salting out” and precipitation, which all rely on dissolving the polymer in an organic solvent [12,13,14,15]. The emulsion-evaporation method involves emulsifying PLGA dissolved in a water-immiscible solvent with water in the presence of a colloidal stabilizer such as poly(vinyl alcohol) (PVA). The emulsification process is usually performed via high energy ultrasonication or homogenization, yielding particles after the subsequent evaporation of the organic solvent. Emulsions can also be stabilized by nanoparticles, a process known as a Pickering emulsion [16], via a reduction in the interfacial energy of the emulsion upon the adsorption of nanoparticles to the emulsion droplet [17,18]. However, this technique has not been widely reported for PLGA particles [19,20,21,22] and has not been explored for bone tissue engineering applications. GO can act as a stabilizer in Pickering emulsions due to its amphiphilic nature [23,24,25], provides a high degree of surface coverage as the GO sheets lay parallel to the droplet interface [26] and can be the sole surfactant in mini-emulsion and emulsion polymerization reactions [27,28,29]. Pickering emulsions possess advantages for biomedical applications, including a high stability, low toxicity and narrow size distribution [30]. 

GO has been combined with PLGA via electrospinning [8,9], freeze drying [31] or emulsion solvent evaporation [11]. This study reports for the first time the preparation of PLGA particles via a Pickering emulsion using GO as the sole surfactant. The particles were not cytotoxic when used at low doses and promoted the osteogenic differentiation of human fetal cartilage rudiment cells in the absence of exogenous growth factors.

## 2. Results and Discussion

### 2.1. Characterization of GO-PLGA Microparticles

**Figure 1:**
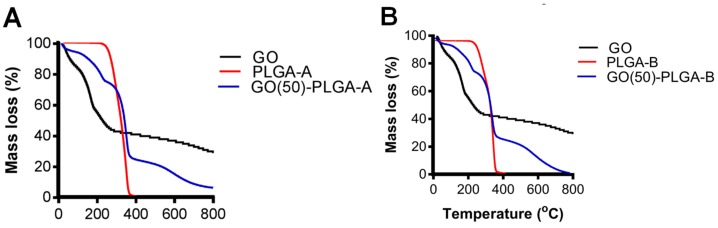
The TGA analysis of the GO (black), (**A**) PLGA-A and GO(50)-PLGA-A and (**B**) PLGA-B and GO(50)-PLGA-B particles presented as mass loss over the temperature range of 20–800 °C.

GO-PLGA particles were prepared via a Pickering emulsion using GO as the stabilizer and anisole as the solvent and were compared to a control system where PLGA particles prepared with PVA as the stabilizer. A thermal gravimetric analysis (TGA) of GO, PLGA and the GO-PLGA particles, prepared with 50 wt% GO, demonstrated a prominent degradation between 300 °C and 400 °C, in accordance with the decomposition of PLGA across this temperature range (Figure 1) [13]. The mass loss of the GO-PLGA particles prepared with 50 wt% GO (GO(50)-PLGA-A) in this temperature range was approximately 50% in agreement with the prepared composition of the dispersion, based on the initial weight fractions of GO and PLGA-A (Figure 1A) or PLGA-B (Figure 1B). Similar results were obtained for PLGA-A with a lower wt% of GO (data not shown).

**Figure 2:**
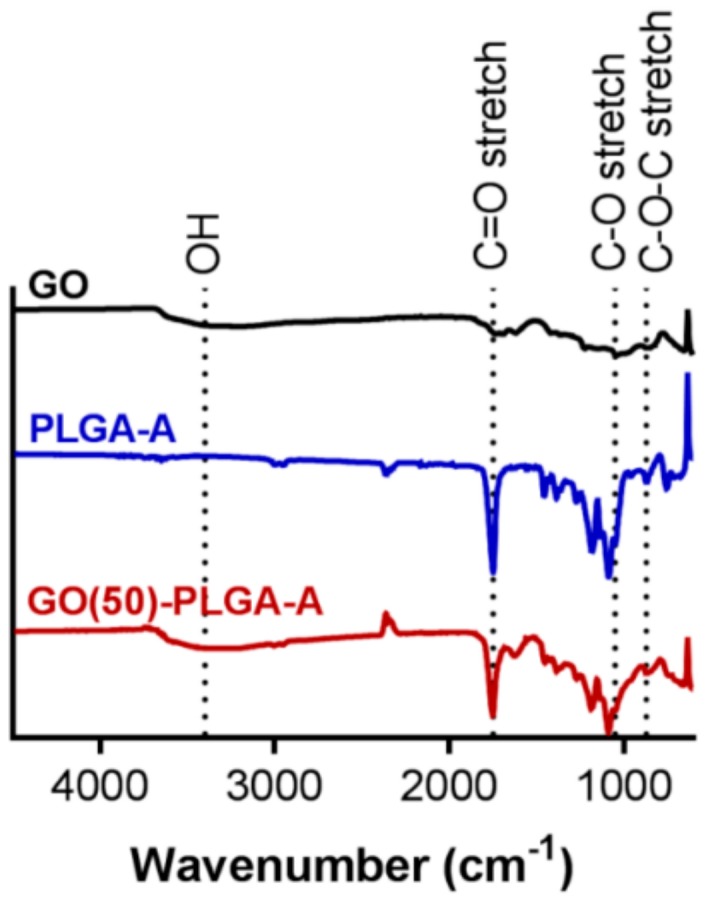
The FTIR spectra of the GO, PLGA-A and GO(50)-PLGA-A particles after freeze-drying, indicating characteristic bands for GO and PLGA.

The analysis of the particle surface bonds by attenuated total reflectance-Fourier transform infrared spectroscopy (ATR-FTIR) confirmed that GO was bound to the PLGA particle surface (Figure 2). GO exhibited a broad stretching peak with a maximum at 3400 cm^−1^ attributed to its large number of hydroxyl groups, as well as absorptions at 1050 cm^−1^ (C–O stretch) and 870 cm^−1^ (C–O–C stretch) attributed to its typical chemical groups [25]. PLGA-A exhibited a strong absorption at ~1750 cm^−1^ indicative of the ester linkage (C=O stretch) within the PLGA backbone. The GO(50)-PLGA-A particles exhibited absorption bands characteristic of both PLGA and GO, further indicating the presence of both species in the particle structure (Figure 2). Similar results were obtained with PLGA-B (data not shown).

**Figure 3:**
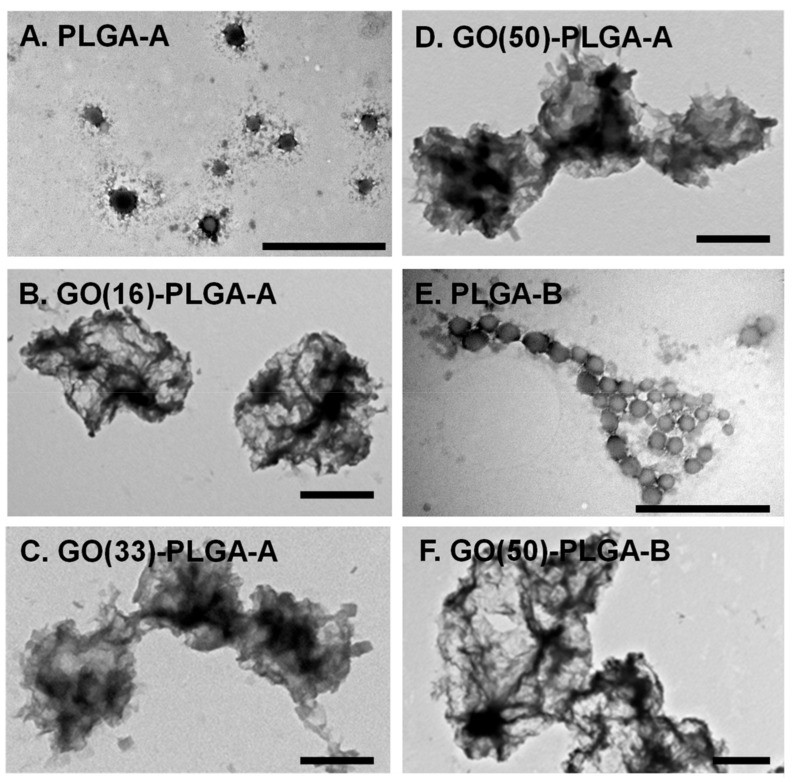
The TEM images of the PLGA and GO-PLGA microparticles prepared with either PLGA-A (**A**–**D**) or PLGA-B (**E**,**F**) without GO (**A**,**E**) or with 16, 33 or 50 wt% GO (denoted GO(16), GO(33) or GO(50)) (**B**–**D**,**F**). Scale bars indicate 1 µm.

**Figure 4:**
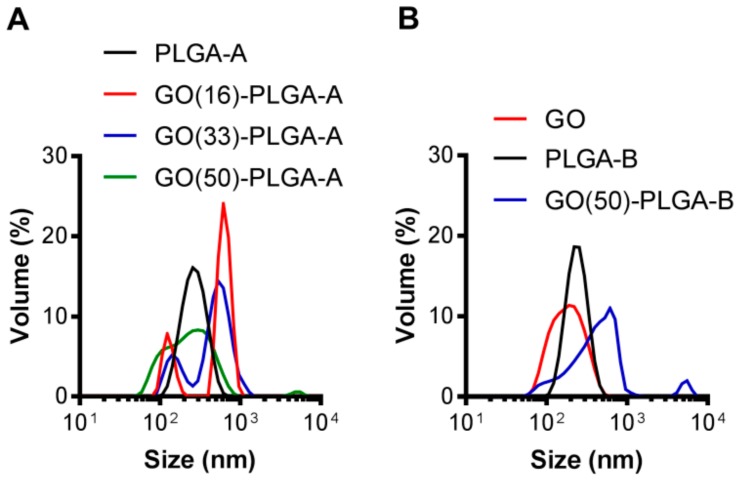
Volume distribution of (**A**) PLGA-A and GO-PLGA-A with different wt% GO; and (**B**) the GO, PLGA-B and GO(50)-PLGA-B particles analyzed by DLS.

The GO:PLGA mass ratio was varied to examine the influence of GO loading on the physical characteristics of the particles. The control PLGA-A and PLGA-B particles formed relatively monodispersed particles of approximately 170 and 125 nm, respectively, determined by transmission electron microscopy (TEM) (Figure 3A,E and Table 1). The largest GO-PLGA-A particles were obtained with 50 wt% GO (GO(50)-PLGA-A) with a size of 1860 ± 350 nm, while GO(16)-PLGA-A were the smallest with a size of 1100 ± 280 nm (Table 1 and Figure 3B,D). The GO(50)-PLGA-B particles were found to have a size of 2350 ± 620 nm (Figure 3F), but were not significantly (*p* < 0.05) different in size to the GO(50)-PLGA-A particles (Table 1). The dynamic light scattering (DLS) analysis also indicated that the incorporation of GO into the particles increased their size (Table 1 and Figure 4); however, there was a large discrepancy between the DLS and TEM measurements of the average diameter (Table 1). This is most likely due to the highly irregular shape of the particles (Figure 3). Additionally, the DLS volume distributions for all samples were broad and bimodal, with the smaller particle diameters attributable to ‘free’ GO in the continuous phase that were not incorporated into the emulsion (Figure 4). The effective hydrodynamic diameter of ‘free’ GO was approximately 120 nm, which is similar in size to pristine GO dispersed in water. Similar bimodal particle size distributions have been reported when GO has been used as a Pickering stabilizer [32]. As the mass fraction of GO in the system decreased, the relative contribution of the ‘free’ GO to the overall particle size distribution also decreased (Figure 4 and Table 1). The zeta potential of all GO-PLGA particles was comparable to the zeta potential of the original GO dispersion (−47.6 mV), further indicative of the fact that GO was adsorbed on the surface of the particles (Table 1).

### 2.2. Cellular Interactions with GO-PLGA Microparticles

**Figure 5:**
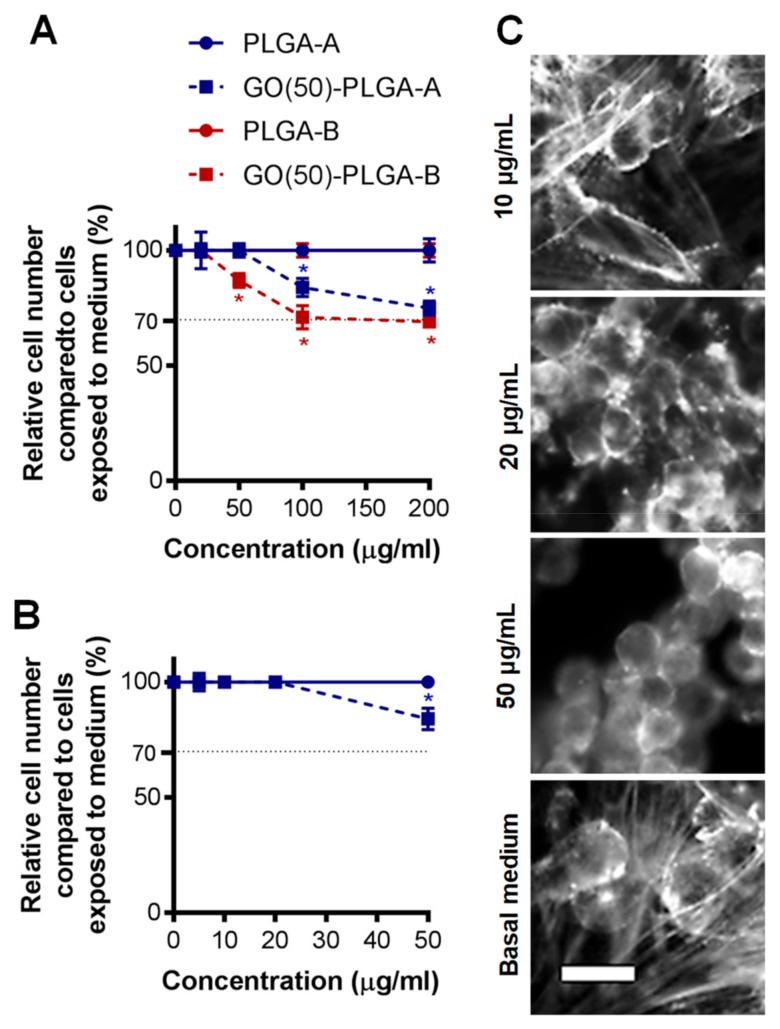
The cytotoxicity of microparticles at concentrations of 0–200 μg/mL exposed to (**A**) human osteoblast-like (Saos-2 cell line) or (**B**) human fetal cartilage rudiment cells after 72 h. Data was normalized to cells grown in the basal medium measured by the MTS assay. Data was presented at mean ± standard deviation (n = 3). * Indicated significant a difference compared to the cells exposed to the basal medium at each time point as determined by a one-way ANOVA, *p* < 0.05; (**C**) The morphology of the human fetal cartilage rudiment cells after 72 h of exposure to GO(50)-PLGA-A at concentrations of 10, 20 and 50 μg/mL compared to the cells grown in the standard medium. The cells were fixed and stained with rhodamine-phalloidin to indicate the actin cytoskeleton. Scale bar represented 20 µm.

As PLGA has been widely investigated for bone tissue engineering applications, it was of interest to determine the effect of the GO-PLGA microparticles on human osteoblast cells. The GO(50)-PLGA-A particles were investigated as they were not appreciably different in size, morphology or surface charge to the other GO-PLGA-A particles and were compared to the GO(50)-PLGA-B particles. Human osteoblast-like cells (Saos-2 cell line) were exposed to particles at concentrations between 0 and 200 µg/mL over a period of 72 h. The control PLGA particles did not reduce the cell number compared to the cells grown in the basal medium at any of the tested concentrations (Figure 5A). In contrast, both the GO(50)-PLGA-A and GO(50)-PLGA-B particles significantly (*p* < 0.05) reduced the cell number at concentrations of 100 µg/mL and greater (Figure 5A), while the 50 µg/mL GO(50)-PLGA-B particles also significantly (*p* < 0.05) reduced the cell number. However, the level of reduction in the cell number was only cytotoxic at GO(50)-PLGA-B particle concentrations of 100 µg/mL and above as there was more than 70% cell growth inhibition. The GO(50)-PLGA-B particles were not analyzed further as they had a lower workable concentration range than the GO(50)-PLGA-A particles without inducing cytotoxicity.

Human fetal cartilage rudiment cells have the potential to express either an osteogenic or chondrogenic phenotype [33] and were used in further studies to determine whether the particles promoted cell differentiation. Human fetal cartilage rudiment cells were exposed to either PLGA-A or GO(50)-PLGA-A particles at concentrations between 0 and 50 µg/mL over a period of 72 h. GO(50)-PLGA-A particles at concentrations between 0 and 20 µg/mL did not change the cell number compared to cells exposed to the basal medium (Figure 5B). In contrast, 50 µg/mL GO(50)-PLGA-A particles significantly (*p* < 0.05) reduced the cell number over the 72 h analysis period compared with cells exposed to the basal medium. However, this reduction in the cell number was not at a level that would be considered cytotoxic as there was not more than 70% cell growth inhibition.

The analysis of the morphology of the human fetal cartilage rudiment cells by staining their actin cytoskeleton indicated that the exposure of the cells to 10 µg/mL GO(50)-PLGA-A had no effect on their morphology compared to cells grown in the basal medium, with cells in both conditions exhibiting a spread and elongated morphology (Figure 5C). In contrast, higher doses of GO(50)-PLGA-A caused the cells to exhibit a rounded morphology (Figure 5C). Thus, further analyses were performed with 10 µg/mL GO(50)-PLGA-A as this was the highest concentration analyzed that did not affect either the number or morphology of the fetal cartilage rudiment cells compared to the cells grown in the basal medium.

**Figure 6:**
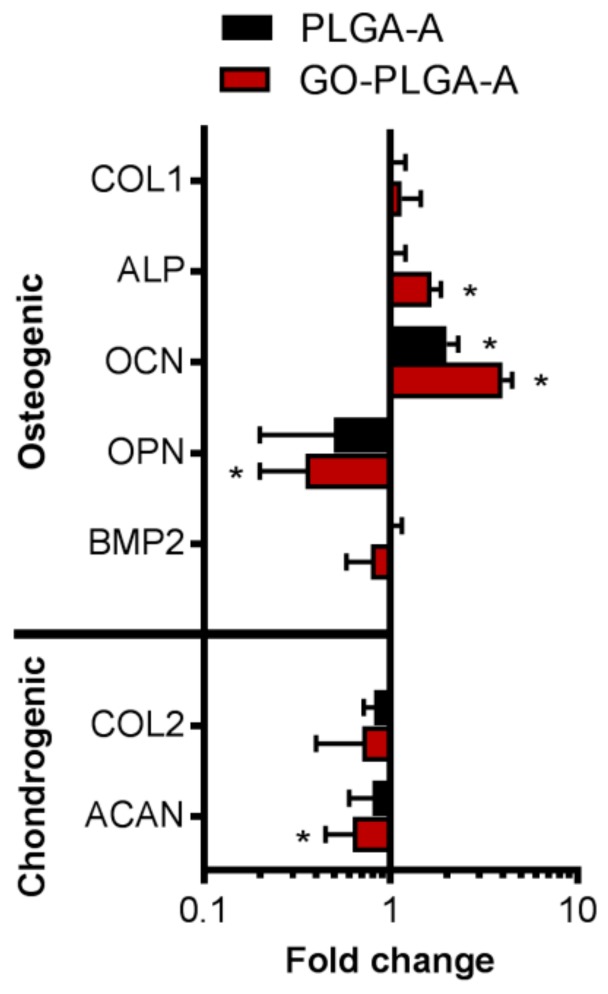
The qPCR analysis of the osteogenic (including collagen type 1 (COL1), alkaline phosphatase (ALP), osteocalcin (OCN) and osteopontin (OPN), bone morphogenic protein (BMP) 2) and chondrogenic (including collagen type II (COL2) and aggrecan (ACAN)) gene expressions by the human fetal cartilage rudiment cells exposed to GO(50)-PLGA-A and PLGA-A at 10 µg/mL compared to the cells grown in the basal medium for 5 days. Data was presented as fold change compared to the cells exposed to the medium only and was corrected for GAPDH expression for each treatment. * Indicated a significant difference compared to the cells exposed to the basal medium as determined by a one-way ANOVA, *p* < 0.05.

The expression of osteogenic and chondrogenic genes by the human fetal cartilage rudiment cells exposed to PLGA-A or GO(50)-PLGA-A for 5 days was analyzed by a quantitative real time polymerase chain reaction (qPCR) (Figure 6). The genes involved in the osteogenic phenotype, including alkaline phosphatase and osteocalcin, were significantly (*p* < 0.05) upregulated in the cells exposed to GO(50)-PLGA-A compared to the cells grown in the basal medium, while only osteocalcin expression was upregulated in the cells exposed to PLGA-A (Figure 6). While collagen type I was upregulated in the cells exposed to either PLGA-A or GO(50)-PLGA-A, it was not upregulated to a level that was considered statistically significant. These data suggest that the GO(50)-PLGA-A particles promoted the osteogenic differentiation of the human fetal cartilage rudiment cells, while PLGA-A had only a weak influence over the short culture time.

Osteopontin is also recognized for its role in bone remodeling, and its expression was significantly (*p* < 0.05) down-regulated in the human fetal cartilage rudiment cells exposed to GO(50)-PLGA-A compared to cells grown in the basal medium; however, there was no significant difference in the osteopontin expression in the cells exposed to PLGA-A compared to the basal medium (Figure 6). An increased expression of osteopontin reduces the osteogenic phenotype expression, reduces the bone morphogenic protein (BMP2) mediated alkaline phosphatase activity and also inhibits cell proliferation [34], and it supported the findings of the present study where GO(50)-PLGA-A promoted the expression of osteocalcin and alkaline phosphatase genes and did not affect cell proliferation. Additionally, gene expression can indicate the stage of osteoblastic differentiation, as alkaline phosphatase and collagen type I are expressed in the initial stages of differentiation whereas osteocalcin is expressed later in the process [35]. Thus, the human fetal cartilage rudiment cells exposed to GO(50)-PLGA-A were committed to the osteogenic phenotype, while cells exposed to PLGA-A were not. These findings are supported by reports of osteogenic gene expression by human mesenchymal stem cells and murine pre-osteoblasts exposed to GO-PLGA alone [10] or incorporated in silk membranes [8,9,10] in the presence of exogenous BMP2. These reports are in line with the known role of BMP2 in promoting osteogenic differentiation [36]. Interestingly, in the present study, BMP2 gene expression by cells exposed to either GO(50)-PLGA-A or PLGA-A was not significantly different to cells exposed to the basal medium (Figure 6), even though other markers of osteogenic phenotype expression were upregulated. This indicates that the level of BMP2 in serum containing a culture medium, together with other factors present in the cultures, was sufficient to promote an osteogenic linage commitment and may be due to the ability of GO to bind biomolecules and concentrate them for presentation to cells [5,6]. While the addition of BMP2 to in vitro cell cultures provides promising results for enhanced bone formation, the clinical application of BMP2 is associated with adverse effects, including ectopic bone formation, bone resorption and unwanted adipogenesis [37]. Thus, materials that can promote the osteogenic phenotype in the absence of exogenous BMP2 are desirable, such as the GO(50)-PLGA-A particles presented here.

Genes involved in chondrogenic phenotype expression, including collagen type II and aggrecan, were downregulated in the human fetal cartilage rudiment cells exposed to GO(50)-PLGA-A compared to the cells exposed to the basal medium, with only aggrecan being downregulated to a level that was considered significant (*p* < 0.05) (Figure 6), suggesting that the GO(50)-PLGA-A did not promote a chondrogenic phenotype. In addition, the human fetal cartilage rudiment cells exposed to PLGA-A did not exhibit a statistically significant change in chondrogenic gene expression. Together, these data suggest that the GO(50)-PLGA-A particles were effective in establishing a human fetal cartilage rudiment cell commitment to the osteogenic lineage in the absence of exogenous osteogenic growth factors. 

## 3. Materials and Methods

### 3.1. Materials

Reagents were purchased from Sigma-Aldrich (Castle Hill, Australia) unless stated otherwise. PLGA with lactide:glycolide ratios of 75:25 (denoted PLGA-A, molecular weight range 66–107 kDa) or 65:35 (PLGA-B, molecular weight range 40–70 kDa), as well as PVA with a molecular weight range 13–23 kDa, were used as received. Graphite nanofibers (Catalytic Materials LLC Ltd, >98%, Pittsboro, NC, USA) were used as received. HCl (Ajax, 32%, Sydney, Australia), H_2_SO_4_ (Ajax, 98%), H_3_PO_4_ (Chem Supply, 85%, Gillman, Australia), KMnO_4_ (Ajax) and H_2_O_2_ (Ajax, 30%) were used as received. Acetone and diethyl ether (reagent grade) were provided by ChemSupply. All water was Milli-Q grade. Human fetal cartilage rudiment cells (composed of chondroblasts/chondroprogenitor cells, as well as committed chondrocytes) were isolated from fetal foot and knee joint cartilage (12–14 week old) in accordance with approval from the Human Research Ethics Committees of UNSW Sydney [38]. The human osteosarcoma cell line, Saos-2, which displays osteoblastic features [39], was purchased from American Type Culture Collection (Manassas, VA, USA). Ultrafrost microscope slides were purchased from Lomb Scientific (Taren Point, Australia). Rhodamine-phalloidin and 4′,6–diamidino–2–phenylindole, dilactate (DAPI) were purchased from Life Technologies, Carlsbad, CA, USA. The RQ1 RNase-Free DNase kit and the MTS reagent were purchased from Promega (Madison, WI, USA). The oligo d(T)23 primer mix (ProtoScript^®^ M-MuLV First Strand cDNA synthesis kit) was purchased from GeneSearch Pty Ltd (Arundel, Australia). The Power SYBR Green PCR Master Mix was purchased from Applied Biosystems (Mulgrave, Australia).

### 3.2. Preparation of Graphite Oxide

Graphite oxide was prepared via the oxidation of graphite nanofibers in a method analogous to that described previously [40]. Briefly, graphite (2 g) was oxidized for 24 h in the presence of KMnO_4_ (graphite:oxidant mass ratio = 1:6) in a 9:1 v/v mixture of H_2_SO_4_ and H_3_PO_4_ (250 mL). At the conclusion of the reaction, the mixture was poured onto ice (~400 g), followed by the dropwise addition of H_2_O_2_ (~10 mL). The graphite oxide product was purified by multiple centrifugation and washing steps, including 3% w/w HCl (3 times), acetone (3 times) and finally with diethyl ether. The product was dried in a vacuum for 24 h at 60 °C and stored in a desiccator until use. The C:O atomic ratio was determined to be 1.37 by an XPS survey scan (ESCALAB™ XI+, ThermoFisher Scientific, Sydney, Australia) (data not shown).

### 3.3. Preparation of GO-PLGA Hybrid Particles

**Figure 7:**
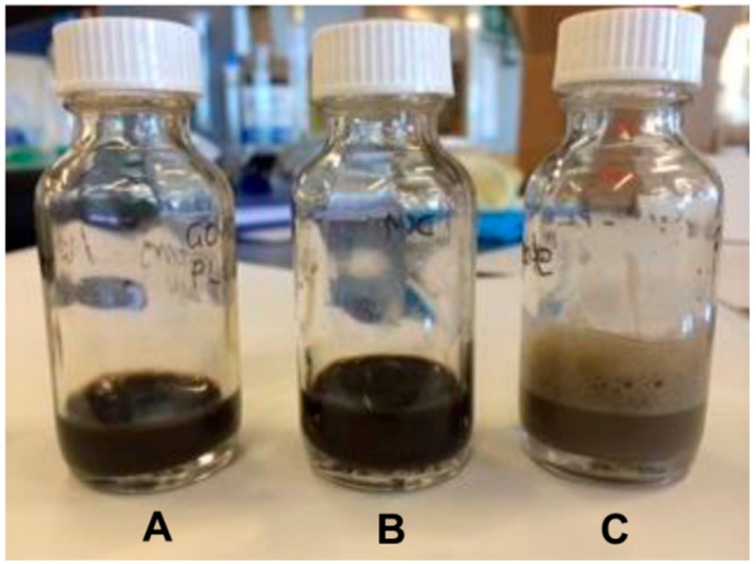
Photograph of the PLGA-A/GO dispersions following ultrasonication for 10 min utilizing different solvents including (**A**) chloroform; (**B**) 1:2 acetone:dichloromethane and (**C**) anisole.

GO was prepared by the exfoliation and dispersion of graphite oxide. Graphite oxide (50 mg) was dispersed in water (9 mL) via ultrasonication (Model 450 Digital Sonifier with microtip, 3 × 20 min cycles, 50% amplitude, 200 W; Branson, Danbury, CT, USA), yielding a stable GO dispersion. The number-average mean diameter of the GO sheets determined by DLS was 140 nm (PDI = 0.15). In a separate vial, PLGA was dissolved at a concentration of 50 mg/mL in either chloroform, acetone:dichloromethane (1:2 ratio by mass) or anisole. Upon dissolution, 1 mL of the PLGA solution was added to the GO dispersion, followed by gentle hand mixing of the two phases and ultrasonication for 10 min on ice (50% amplitude). The resultant mini-emulsion was observed with respect to colloidal stability for ~24 h, followed by dialysis (using regenerated cellulose dialysis membranes, MWCO = 3500 Da) against water for four days to remove the organic solvent. Chloroform and acetone:dichloromethane did not result in the formation of a stable emulsion, while anisole did and was thus used as the solvent to prepare the particles (Figure 7). 

In certain experiments, the PLGA:GO mass ratio was varied to examine the influence of GO loading. Freeze drying was used to isolate and analyze the PLGA-GO composite material. In order to prepare the PLGA nanoparticles in the absence of GO, an analogous procedure was used; however, PVA (50 mg) was dissolved in water (9 mL) in lieu of the GO dispersion. PLGA (50 mg) was dissolved in 1 mL of a 1:2 w/w acetone:dichloromethane mixture. The two solutions were mixed and then ultrasonicated using identical conditions to those above, followed by dialysis against water.

### 3.4. Characterization of Particles

Hydrodynamic diameter and zeta potential measurements were performed by DLS (ZetaSizer Nanoseries with DTS software, Malvern, UK), utilizing a 4 mW He-Ne laser at 633 nm at a backscattering angle of 173°. The values are reported as an average of three independent measurements, performed on a diluted sample (1 drop of the nanoparticle dispersion in 20 mL water). The ATR-FTIR spectra were recorded on a IFS66-S instrument (Bruker, Billerica, MA, USA) with a diamond plate, tungsten halogen lamp and calcium fluoride beam splitter. 64 scans were recorded per sample over the wavenumber range of 500–4000 cm^−1^ (4 cm^−1^ resolution). X-ray photoelectron spectroscopy (XPS) was performed on an Escalab 250Xi spectrometer (Thermo Scientific, Loughborough, UK). The incident radiation was Al X-rays (1486.6 eV) at 150 W (12 kV, 12 mA). A 165 mm hemispherical electron energy analyzer was used, with survey scans taken at an analyzer pass energy of 100 eV over a 1360–0 eV binding energy range (1.0 eV steps, dwell time 100 ms). Higher resolution scans were taken at a 20 eV analyzer pass energy (0.1 eV steps, dwell time 250 ms). The base pressure in the analysis chamber was below 0.2 μPa, and below 1.3 μPa during the analysis. The particle analysis was performed on a JEM-1400 TEM (JEOL, Peabody, MA, USA) at an accelerating voltage of 100 kV. The specimen preparation consisted of the casting of a drop of diluted aqueous dispersion onto a copper-Formvar grid, then allowed to dry at room temperature. TGA was performed on a Q5000 instrument (TA Instruments, New Castle, DE, USA) by heating the sample to 800 °C at a heating rate of 10 °C/min. The atmosphere was either nitrogen or air at a flow rate of 2 mL/min.

### 3.5. Culture of Cells

The human osteoblast-like cell line (Saos-2) and the human fetal cartilage rudiment cells were cultured in Dulbecco’s modified eagle culture medium supplemented with 2 mM L–glutamine, 50 µg/mL L–ascorbic acid to promote extracellular matrix synthesis [41], 1% (v/v) penicillin/streptomycin and 10% (v/v) fetal calf serum and used for experiments at passage 2.

### 3.6. Cell Viability

Cells, either the human osteoblast-like cell line (Saos-2) or the human fetal cartilage rudiment cells, were seeded in 96-well tissue culture polystyrene plates at a density of 10^4^ cells/well in a 200 µL medium. The cells were incubated for 4 h prior to the addition of the particles at concentrations 0–200 µg/mL. The cell viability was analyzed at 72 h after the addition of particles using the MTS assay. The MTS reagent was added to the cell cultures 6 h prior to the measurement of the absorbance at 490 nm, and material only controls were used to determine that the particles did not interfere with the MTS reagent.

### 3.7. qPCR

The total RNA was isolated from the human fetal cartilage rudiment cells using TRI Reagent (1 mL per 10^7^ cells) and then treated with DNase using the RQ1 RNase-Free DNase kit to remove contaminating DNA. Subsequently, 1 µg RNA was transcribed into cDNA using an oligo d(T)23 primer mix (ProtoScript^®^ M-MuLV First Strand cDNA synthesis kit). For quantitative real-time PCR (qPCR), 1 µL cDNA was mixed with 0.5 µL forward and reverse primers (10 µM each) and 10 µL Power SYBR Green PCR Master Mix, followed by adding RNAase free water to make up to 20 µL per sample. The primers include collagen type II (F: 5′–ACACTGCCAACGTCCAGATGAC–3′ and R: 5′–CAGTGTACGTGAACCTGCTATTGC–3′), aggrecan (F: 5′–TGTCCAAGGAGAAGGAGGTAGTG–3′ and R: 5′–GATGCCTTTCACCACGACTTC–3′), collagen type I (F: 5′–TAAAGGGTCACCGTGGCTTCTC–3′ and R: 5′–GCAGGAAGCTGAAGTCGAAACC–3′), alkaline phosphatase (F: 5′–AGGGCTGTAAGGACATCGCCTA–3′ and R: 5′–GCGGTTCCAGATGAAGTGGGA–3′), osteocalcin (F: 5′–CGAGGTAGTGAAGAGACCCA–3′ and R: 5′–GTGGTCAGCCAACTCGTCAC–3′), osteopontin (F: 5′–CATGTGGACAGCCAGGACTC–3′ and R: 5′–TGTGAGGTGATGTCCTCGTC–3′), bone morphogenic protein 2 (BMP2) (F: 5′–GGTTCAACCCCAGCACATGAAGT–3′ and R: 5′–GCTGCGTGTTGGGCAAAAAGT–3′) and GAPDH (F: 5′–AGAAGGCTGGGGCTCATTTG–3′ and R: 5′–AGGGGCCATCCACAGTCTTC–3′). GAPDH was used as an endogenous control and to normalize the results for each of the primer sets. The samples were subjected to 40 reactions using the ABI StepOne™ Real-time PCR system.

### 3.8. Statistical Analyses

A one-way analysis of variance (ANOVA) was conducted with a Tukey’s post-hoc test to compare multiple conditions after establishing that the data were normally distributed. Results of *p* < 0.05 were considered significant. Cell experiments were performed in triplicate.

## 4. Conclusions

This study demonstrated that GO-PLGA microparticles can be synthesized via a Pickering emulsion using GO as the sole surfactant. The size of the particles produced was stable in the range of 1.1 to 2.4 µm when GO was used in the range of 16–50 wt%. This study demonstrated that neither PLGA nor GO-PLGA microparticles were cytotoxic to either human osteoblast-like or human fetal cartilage rudiment cells when used at doses of up to 50 µg/mL. The GO(50)-PLGA-A microparticles supported an osteogenic linage commitment in human fetal cartilage rudiment cells in the absence of exogenous growth factors. In contrast, while human fetal cartilage rudiment cells exposed to PLGA expressed osteogenic genes, they were not committed to this linage over the analysis period. Together, these data suggest that the incorporation of GO into PLGA particles is a promising approach for an ‘off-the-shelf’ biomaterial capable of promoting rapid bone regeneration in the absence of expensive and rapidly degraded growth factors.

## Figures and Tables

**Table 1:** The PLGA and GO-PLGA particle size and charge determined by TEM, DLS and zeta potential.

PLGA	PLGA Lactide: Glycolide Ratio	GO wt %	TEM Diameter (nm)	DLS Diameter/PDI (nm)	Zeta Potential (mV)
A	75:25	0	170 ± 50	236/0.12	−32.4
		16	1100 ± 280	734/0.64	−42.2
		33	1220 ± 340	506/0.50	−48.2
		50	1860 ± 350	270/0.27	−46.6
B	65:35	0	125 ± 20	219/0.06	−25.3
		50	2350 ± 620	456/0.52	−40.3
